# Heart rate variability change during a stressful cognitive task in individuals with anxiety and control participants

**DOI:** 10.1186/s40359-021-00551-4

**Published:** 2021-03-17

**Authors:** Judith Held, Andreea Vîslă, Christine Wolfer, Nadine Messerli-Bürgy, Christoph Flückiger

**Affiliations:** 1 Department of Psychology, Psychological Interventions and Psychotherapy, University of Zurich, Binzmühlestr. 14/04, 8050 Zurich, Switzerland; 2 Department of Psychology, Clinical Child Psychology and Biological Psychology, University of Freiburg, Freiburg, Switzerland

**Keywords:** Heart rate variability, Anxiety, Stress, Working memory, Worry

## Abstract

**Background:**

Individuals suffering from an anxiety disorder are characterized by chronically low heart rate variability (HRV) compared to healthy individuals during resting state conditions. However, when examining HRV and HR in response to a stressor, mixed results have been obtained when comparing anxious and non-anxious groups.

**Methods:**

The primary aim of the present study was to investigate HRV and HR responding in 26 clinically anxious and 14 control individuals before, during and after a stressful working memory task.

**Results:**

Results indicate no between-group differences in HRV and HR at baseline. When starting the working memory task, the control group decreased significantly in HRV and the anxious group did not differ substantially in their change pattern from baseline to the start of the stressor. Finally, during the recovery phase of the working memory task, the clinically anxious and control individuals did not differ in their HFV or HR response compared to baseline.

**Conclusions:**

From a clinical perspective, the results suggest that screening for the presence of anxiety disorders may help to identify patients with impaired HRV and HR functioning and to intervene on these important patient characteristics early in the treatment process.

**Supplementary Information:**

The online version contains supplementary material available at 10.1186/s40359-021-00551-4.

## Background

Anxiety disorders have been frequently found to be associated with distorted cardiovascular activity [1, 2]. The most commonly investigated measure of cardiovascular activity in anxiety disorders are heart rate variability (HRV) and heart rate (HR) where HRV indicates the degree to which the autonomic nervous system adapts to environmental and situational demands through regulating cardiovascular processes [3]. The high frequency component of HRV (HF-HRV) has been linked to parasympathetic influences of the autonomic nervous system [4], which are thought to be responsible for producing rapid changes in heart beats, necessary to adapt to environmental demands. A growing body of literature suggests a reduced activation of the autonomic nervous system in anxiety disorders resulting in decreased HRV, e.g., by chronically low HRV during resting state conditions [5]. However, findings regarding the HRV response in anxious individuals in reaction to a stressor are mixed [2, 6]. Therefore, the present study seeks to investigate HRV in anxious compared to non-anxious individuals before, during and after a stressor.

The generalized unsafety theory of stress (GUTS) provides a possible explanation of how decreased HRV and anxiety disorders might be linked [7]. It proposes that the stress response is per default active but chronically inhibited as long as safety is perceived. When a threat or stressor is perceived, this cognitive inhibition is removed triggering the default stress response which results in physiological activation (e.g., increase in HR, decrease in HRV). When the stressor has ended, the stress response is again inhibited and physiological activation returns to “normal” values. However, according to GUTS, chronically anxious individuals have difficulty detecting safety and therefore, the chronic stress response is not inhibited but rather stays chronically active [5]. The GUTS differentiate between the stress response during a stressor and the prolonged stress response, namely the recovery after the stressor has ended [8]. According to GUTS, the prolonged stress response from experimental stressors is associated with adverse effects of anxiety and stress; e.g., by an increased risk of cardiovascular disease [9]. Stated differently, an anxious individual does not seem to recover from the stress response but still chronically stressed (e.g., [10]).

In line with this notion, the majority of studies examining HRV at rest found that anxiety disorders are associated with chronically low HF-HRV [1]. More specifically, chronically low HF-HRV has been consistently found in generalized anxiety disorder (GAD; 6), social anxiety disorder [11] and panic disorder [12], compared to healthy individuals [2]. In a meta-analysis, the authors summarizing studies that compared resting HRV levels in anxiety disorder patients and healthy controls, the authors found an overall small to moderate negative associations between anxiety disorders in general and reduced HRV (Cohen’s *d* = -0.29; *k* = 36; 1). Taken together, anxiety seems to be marked by chronic physiological responses that are inappropriate to current environmental demands and the stress response seems to be triggered even when no apparent threat is evident [8].

Interestingly, experimental studies investigating HRV in response to a stressor in anxious and non-anxious populations obtained somewhat mixed results. When examining the stress response *within* a specific anxiety disorder group, studies consistently reported a decrease in HRV when confronted with the stressor compared to resting conditions (e.g., [6, 13]). Stressors included being exposed to anxiety-provoking tasks [2, 14], performing cognitive tasks [10, 15, 16] and engaging in worry [6, 17, 18].

Furthermore, when examining the stress response *between*-groups (i.e., anxiety disorders vs. control groups) studies reported mixed results (e.g., [2]). For example, Hammel and colleagues [19] found that GAD and non-GAD control participants (without current diagnosis of GAD, panic disorder or depression) did not differ significantly in HF-HRV indices at rest, during worry or a cognitive challenge. However, the authors obtained a significant main effect of condition: Overall, both groups showed significantly lower HF-HRV during worry compared to the resting condition. Moreover, Pittig and colleagues [2] found significantly lower HF-HRV during a hyperventilation task in patients with panic disorder as well as in patients with obsessive compulsive disorder compared to healthy controls. Patients with panic disorder and GAD demonstrated greater HR than healthy controls during hyperventilation. However, patients with GAD and social anxiety disorder did not show significantly different HF-HRV during hyperventilation compared to healthy controls. Taken together, when examining the immediate response to a stressor, mixed results have been reported for anxious and non-anxious individuals.

To summarize, anxiety seems to be marked by chronic physiological stress responses that are inappropriate to current environmental demands. However, when examining the immediate stress response, mixed results have been obtained when comparing anxious and non-anxious groups. Interestingly, although a range of different stressors has been used (i.e., hyperventilation task, cognitive task), the mixed results do not seem to be solely linked to the variety of tasks (e.g., [6, 15]) but certain patient characteristics (i.e., disorder group, 2). Moreover, there seems to be a lack of studies investigating the HRV and HR response before, during and after a stressor within one study; rather, many studies focused on one phase of the stress response (e.g., [10]). Therefore, the present study seeks to investigate the HRV and HR response in individuals with an anxiety disorder compared to control individuals before, during and after performing a stressful working memory (WM) task. To adjust for potential confounds, we considered measures of symptom severity (worry), as well as age and gender in the analyses [2, 11].

Based on the literature above, we formulated the following hypotheses:

### Hypothesis 1

(Before stressor) We expect anxious individuals to have lower HF-HRV and higher HR than control individuals at baseline (e.g., [1]).

### Hypothesis 2

(During stressor) We hypothesize that both anxious and control participants will decrease in HF-HRV and increase in HR when confronted with a stressor (start of the WM task), compared to the baseline phase (e.g., [6, 15]).

### Hypothesis 3

(After stressor) We expect stable, unaffected low HF-HRV level and high HR in anxious participants during the recovery phase, in comparison to control participants where we expect an increase in HRV and a decrease in HR [10].

### Hypothesis 4

We aim to explore if potential differences in HF-HRV and HR are impacted by relevant demographic variables (i.e., baseline worry, age and gender).

## Methods

### Participants

Thirty-three individuals meeting diagnostic criteria for a current anxiety disorder and 22 control subjects were recruited. The study was approved by the Ethical Committee of Canton Zurich (BASEC 2016-00773). Clinically anxious participants were recruited as part of a larger randomized clinical trial (RCT) for cognitive-behavioral therapy for generalized anxiety disorder (GAD) patients [20]. In line with the recommendations of the Task Force of the European Society for rigorous HRV data quality [4], a total of 15 individuals (Anxious group: 7; Control group: 8) were excluded from data analysis due to insufficient HRV data quality.

The total sample included in this study consisted of 26 clinically anxious individuals (20 females; *M*_age_ = 27 years, *SD* = 8.4; Body Mass Index *M*_BMI_ = 21.5, *SD* = 2.8) and 14 non-anxious control subjects (12 female; *M*_age_ = 25.31 years, *SD* = 5.78; Body mass index *M*_BMI_ = 21.6, *SD* = 4.2; Additional file 1: Table S1). The total sample was German speaking and age, gender, nationality, socio-economic status and body mass index did not differ significantly between the groups. Participants did not report any history of a chronical medical condition (i.e., cardiovascular disorders, diabetes, hypertension, hypothyroidism or hyperthyroidism).

Current mental disorder in the anxious group were assessed with the Structural Clinical Interview [21] of the Diagnostic and Statistical Manual of Mental Disorders [22]. The anxious group was comprised of individuals meeting diagnostic criteria for generalized anxiety disorder (GAD; *n* = 14), panic disorder (*n* = 7), specific phobia (*n* = 2), obsessive–compulsive disorder (*n* = 1), panic disorder with agoraphobia (*n* = 1) and agoraphobia (*n* = 1). Furthermore, six individuals had a further comorbid mental health disorder. Control participants were students from the University of Zurich (Switzerland) and were recruited during university courses in exchange for course credit.

Clinical symptoms assessed prior to the working memory (WM) task differed significantly between anxious and control participants. Worry severity, assessed with the Penn State Worry Questionnaire (PSWQ, [23]) was significantly higher in the anxious group compared to the control group (Anxious group: *M* = 64.4, *SD* = 7.4; Control group: *M* = 45.6, *SD* = 7.1; *F* (1, 37) = 59.8, *p* > 0.001). These results confirm the grouping of the anxious and control individuals; meaning that the anxious individuals were more anxious at baseline (i.e., in expectation to perform a working memory task based on information provided in the informed consent form).

### Procedure

All of the participants performed the WM task under comparable conditions. Participants performed a WM task consisting of two Blocks (Block 1 and 2) and rated their current worry level at three time points (see Additional file 1: Fig. S1). First, in an initial warm-up phase, participants filled out the written informed consent form for study participation and the PSWQ. Then, participants attached the HRV sensor and were seated upright. A three-minute sitting phase followed to collect baseline HRV and HR measures (“baseline”). Afterwards participants were asked to rate their current level of worry. Next, the WM task was explained verbally by the experimenter. Participants completed two practice trials and afterwards, WM Block 1 started (“WM task”). After the first WM Block, participants rated their current level of worry and started WM Block 2. After the completion of WM Block 2, participants were again asked to rate their level of worry. Finally, a three-minute recovery period followed (“recovery”).

### Questionnaires

The Penn State Worry Questionnaire (PSWQ, [23]) is a 16-item self-report questionnaire assessing pathological worry in clinical and non-clinical populations. Answers are given on a 5-point Likert scale, ranging from 1 (“not at all typical for me”) to 5 (“very typical of me”). Internal consistency in the present study was good (Cronbach`s *α* = 0.89).

At three time points (see Additional file 1: Fig. S1), the current level of worry was assessed with a visual analogue scale ranging from 0 to 100 (0 = no worry to 100 = extreme worry), a self-administrated, time-ecological measure that has been investigated across various fields (e.g., Bijur, Silver, & Gallagher, 2001). Descriptively, the highest level of worry in both groups was reported at baseline (Anxious group: *M* = 43.6, *SD* = 21.7, Control group: *M* = 26.1, *SD* = 18.9) with the anxious group reporting significantly higher worry at baseline (*t* (37) = 2.53, *p* = 0.015). I correlational table of the investigated variables is documented in the Additional file 1: Table S2.

#### Working memory task

A numerical updating WM task [24] was used with two repeated WM blocks each lasting on average 10 min (for a detailed description of the task see Additional file 1:). The WM task was self-paced and the duration of each block differed between participants.

### HRV Data Recording and Processing

Physiological data was recorded with the movisens ECG Move 3 sensor (movisens, Karlsruhe, Germany), an ambulatory monitoring system to collect high-quality ECG data. The sensor was attached with two disposable electrodes on the left chest and ECG data was sampled continuously at 1024 Hz. The raw Electrocardiogram (ECG) data was visually inspected and divided into relevant segments using the Unisens viewer software (http://unisens.org/index.php). For the present study, the first three minutes of the WM task were analyzed to investigate HRV and HR responding when initially being confronted with WM task. Therefore, the segments of interest included the 3-min baseline phase, the first 3-min segments of the WM Block 1, and a 3-min recovery period, resulting in three segments (Baseline, WM task, recovery). ECG data was visually inspected for artifacts and artifacts were removed. ECG data was imported in Kubios HRV 3.0 software [25] to calculate inter-beat intervals (IBI) and to calculate HRV parameters. A smooth priors detrending method (λ = 500) was applied to detrend inter-beat time series. In line with the recommendations of the Task Force of the European Society [4], frequency domain measures were calculated by Fast Fourier Transform using Welch`s Periodogram (window width 300 s, 50% overlap, resampled at 4 Hz). Physiological metrices heart rate (HR) in beats per minute and high frequency heart rate variability (HF-HRV) are reported in raw HF units (log transformed) and normalized units. Normalized units were used for the statistical analyses.

### Data analysis

In order to address Hypothesis 1 to 4, multilevel modelling approach was applied in order to address the interdependence of the repeated measurements of HF-HRV and HR [26]. All statistical main analyses were performed in R statistical software [27] using the “nlme” package [28] und the “multilevel” package [29]. Multilevel modelling was performed where time (at Level-1) was nested within patients (at Level-2). For time, the baseline phase was coded with “0” (representing the reference time point), WM task was coded with “1” and the recovery phase was coded with “2”. Furthermore, anxious vs. control group was included as a level-2 individual characteristic, with the control group representing the reference group (coded as “0”). HF-HRV and HR were investigated as dependent variables. To address Hypothesis 4, PSWQ, age and gender were grand-mean centered and entered as covariates into the model.

With a sample of 26 and 14 participants clustered in two groups and an alpha level of 0.05 and a power of 0.80, we are able to reliably detect a large effect size difference of *Cohen’s d* = 0.84 [30]*.*

## Results

**Hypothesis 1** (Before stressor)

**Fig. 1:**
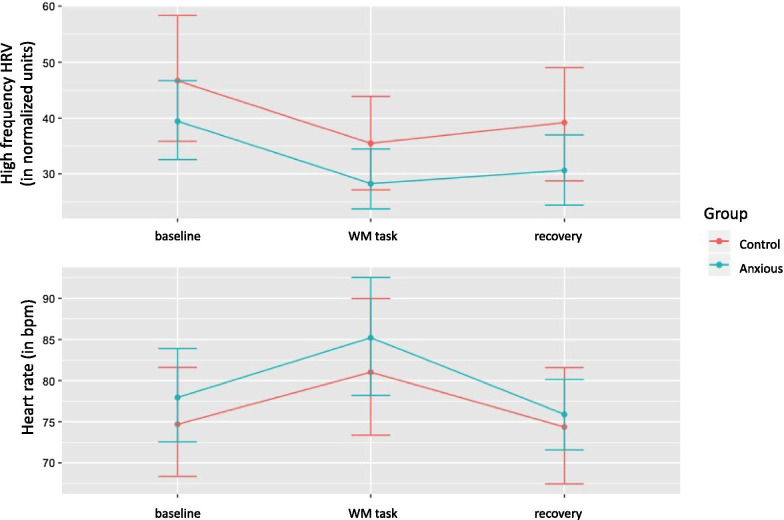
High frequency heart rate variability in normalized units and heart rate over the course of the experiment. Note. *HRV* heart rate variability, *bpm* beats per minute, *WM* working memory. Error bars represent standard error

 Descriptively, mean HF-HRV and HR at baseline were lower in the anxious group (HF-HRV: log transformed units *M* = 6.82, *SD* = 1.0 and normalized units *M* = 39.4, *SD* = 18.9; HR: *M* = 77.9, *SD* = 15.0) than in the control group (HF-HRV: log transformed units* M* = 6.74, *SD* = 1.42 and normalized units *M* = 46.7, *SD* = 22.4; HR: *M* = 74.7, *SD* = 13.2). However, in the multilevel models group differences reveald non-significant for baseline HF-HRV (*t* (38) =  −1.08, *p* = 0.28) and baseline HR (*t* (38) = 0.68, *p* = 0.49; Fig. 1). Therefore, Hypothesis 1 was not confirmed.

**Hypothesis 2** (During stressor)

 For Hypothesis 2, we investigated how anxious and control participants responded when being confronted with a stressful working memory task by comparing the *change* from baseline in HF-HRV and HR within participants. For the control group, results indicated a significant decrease in HF-HRV (*t* (76) =  −2.35, *p* = 0.02) as well as a significant increase in HR (*t* (76) = 3.26, *p* = 0.002) from baseline to WM task (Fig. 1). Of note, when looking at the time by group interaction, no significant differences were obtained for HF-HRV (*t* (76) = 0.01, *p* = 0.99) and for HR (*t* (76) = 0.38, *p* = 0.70), meaning that the groups did not differ substantially in their change pattern from baseline to WM task. Therefore, Hypothesis 2 was partly confirmed as both groups indicated a significant change.

**Hypothesis 3** (After stressor)

 To investigate how anxious and control participants recover after a stressor has ended, HF-HRV and HR in the baseline phase were compared to HF-HRV and HR values obtained in the recovery phase. The results indicated no significant differences in HF-HRV (*t* (76) =  − 1.65, *p* = 0.10) and HR (*t* (76) =  −0.23, *p* = 0.81) for the control group between the baseline and the recovery phase. The time by group interaction was not significant.^1^

**Hypothesis 4** (Impact of PSWQ, Age and Gender) There was no significant main effect of PSWQ, age or gender on HF-HRV and HR at baseline or in the recovery phase (all *p* > 0.05). For the recovery phase, the time by group interaction (*t* (72) =  −2.23, *p* = 0.028) reached significance when gender was controlled for, i.e., the anxious group demonstrated a significant decrease in HF-HRV from baseline to recovery phase. Additionally, a significant time by gender interaction (*t* (72) =  −2.62, *p* = 0.01) indicated that female participants showed a significant decrease in HF-HRV from baseline to recovery. Finally, a significant time by group by gender interaction (*t* (72) = 2.26, *p* = 0.026) was obtained. Besides these effects, no further interaction revealed significance when integrating PSWQ, age and gender into the models.

## Discussion

Individuals suffering from an anxiety disorder are characterized by chronically low heart rate variability (HRV) compared to non-anxious individuals during resting state conditions [1]. However, when examining HRV in response to a stressor, there is mixed evidence about potential between-group differences in clinically anxious vs. non-anxious populations [2, 19]. Therefore, using a repeated measures design with a stressful working memory (WM) task, the primary aim of the present study was to investigate high frequency heart rate variability (HF-HRV) and heart rate (HR) responding in anxious and control individuals before, during and after this cognitive stressor.

In Hypothesis 1, we investigated HF-HRV and HR differences at resting baseline phase in anxious participants suffering from a current anxiety disorder compared to control participants. Contrary to our expectations, we did not obtain significant group differences in HF-HRV or HR which does not seem to be in line with the vast majority of studies [1, 31]. However, when taking a closer look, there are a handful of studies that did not obtain significant resting-state differences in HF-HRV in anxiety disorder patients vs. control participants [17, 19]. There are various potential explanations for the discrepancy in findings. First, our anxious group was composed of different primary anxiety disorders, and it is possible that the relation between reduced HF-HRV and HR varies across anxiety disorders. Indeed, there is evidence that some anxiety disorders are associated with stronger decreases in HF-HRV than others [1, 32]. Secondly, there may be shared characteristics common to all anxiety disorders besides the mere clinical diagnosis (i.e., transdiagnostic mechanisms) which are better able to capture cardiovascular differences compared to healthy participants. One potential transdiagnostic factor is worry and, there is some evidence that worry may be more consistently associated with reductions in HRV [17, 33].

Next, we investigated the initial HF-HRV and HR response to a stressor (Hypothesis 2) and as hypothesized, confrontation with a stressor resulted in a decrease in HF-HRV and in increase in HR in the control group. Moreover, groups did not differ significantly and there was no time by group interaction. Interestingly, the results of the self-reported level of worry was highest in both groups at baseline (assessed after the baseline phase and before the first WM Block) and the anxious group reported significantly higher worries than the control group at baseline. Therefore, whereas the cardiovascular indices showed relatively higher vagal tone in the baseline condition compared to the stress condition, the self-reported level of worry was highest between baseline and stressor initiation. One possible explanation for this discrepancy is that the mere announcement of the WM task itself triggered a stress response resulting in higher worry after baseline HRV recording and before the start of the WM task. In line with this notion is the finding that HF-HRV was significantly lower and HR significantly higher at the start of the stressing WM task compared to baseline condition, therefore it seems reasonable that at baseline, participants were not as stressed as during the WM task. Taken together, we obtained preliminary evidence that the stress response seems to be a more universal response independently of having a current anxiety disorder diagnosis or not [3] and initial baseline cardiovascular activity.

Besides the magnitude, the duration of the stress response may be an important indicator of post stress recovery [34]. Therefore, in Hypothesis 3, we examine HF-HRV and HR responding during the recovery phase of the anxious versus control individuals [10]. Contrary to our prediction, the anxious and control group did not differ in their HF-HRV and HR values in the recovery phase, compared to baseline. Therefore, Hypothesis 3 was not confirmed. Interestingly, when we controlled for gender, the two groups differed significantly in their HF-HRV change from baseline through recovery phase. These results point in a similar direction as Weber and colleagues’ findings 2010) which indicate different recovery patterns for individuals with high and low HRV [10]. A potential explanation for the discrepancy in findings may be that Weber et al. [10] only used male participants whereas our sample was predominantly female. More specifically, gender may have partially impacted our results, as shown in the significant time by gender interaction for HF-HRV.

Several limitations of the current study are important to note. First, our control group was small (*n* = 14) primarily due to technical constraints of the HRV sensors and subsequent loss of 36% of the data (20% in the anxious group). However, HRV data loss is not uncommon in cardiovascular research [35]. Importantly, when data was lost, this affected the whole data set of a participant and not just a single segment. Second, the overall sample size was small and power calculations indicated that the sample size would be able to detect large effects. Even though the present sample size is comparable to prior studies (*N* = 35, 24, 44 see [10, 16, 19]) larger samples would be preferred. Thirdly, we did not adjust for respiration parameters, such as respiration frequency and depth as suggested by Laborde [36]. However, respiration and HRV oscillations may share the same origins [37] under very low and high breathing conditions which cannot be expected in a upright sitting position [36]. Finally, the anxious group was comprised of individuals with various anxiety disorders, which might have affected the study results [32]. Of note, PSWQ scores were comparable with scores reported in other studies using clinical groups and control groups [18, 19, 38].

## Conclusions

This is one of the first studies that systematically investigated cardiovascular responding before, during and after a cognitive stressor (i.e., a WM task) in clinically anxious and control participants. The obtained results indicate different pattern of cardiovascular activity before and during the experiment.

## Supplementary Information

**Additional file 1: Additional information on methods:** Participants, Working memory task. **Table S1:** Demographic characteristics of the included participants. **Figure S1:** Experimental procedure. **Additional results:** Analysis of the self-reported level of worry across the WM task. **Table S2:** Bivariate correlations.

## Data Availability

The data will be available from the author upon reasonable request. The raw data will not be publicly available because it contains information that could compromise the participant’s privacy. An overview of the output data is available at: http://p3.snf.ch/project-163702.

## Abbreviations

HR: Heart rate
HRV: Heart rate variability
HF-HRV: High frequency component of heart rate variability
GUTS: Generalized unsafety theory of stress
GAD: Generalized anxiety disorder
WM: Working memory
RCT: Randomized clinical trial
PSWQ: Penn State Worry Questionnaire
ECG: Electrocardiogram

## Footnotes

1 In order to check the stability of our results, we recoded the groups with the anxious group being the reference group. **Hypothesis 1 (before stressor)**: Again, we did not find significant differences between the groups in baseline HF-HRV (*t* (38) = 1.08, *p* = .28) and baseline HR (*t* (38) =—0.68, *p* = .49). **Hypothesis 2 (during stressor):** For the anxious group, results indicated a significant decrease in HF-HRV (*t* (76) = -3.19, *p* = .002) as well as a significant increase in HR (*t* (76) = 5.11, *p* > .001) from baseline to WM task. The time by group interaction was not significant. **Hypothesis 3 (after stressor)**: The results indicated significant differences in HF– (*t* (76) = -2.65, *p* = .009) for the anxious group between the baseline and the recovery phase. HR was not significantly different in the anxious group between the baseline and the recovery phase (*t* (76) = -1.96, *p* = .053). The time by group interaction was not significant.
