# Supplementary material for: Heart rate variability change during a stressful cognitive task in individuals with anxiety and control participants
Source: BMC Psychol. 2021 Mar 17;9:44. doi: 10.1186/s40359-021-00551-4 (PMC7972344; doi:10.1186/s40359-021-00551-4)
Supplement: Supplementary file 1 — Additional file 1: Additional information on methods: Participants, Working memory task. Table S1: Demographic characteristics of the included participants. Figure S1: Experimental procedure. Additional results: Analysis of the self-reported level of worry across the WM task. Table S2: Bivariate correlations. [file 40359_2021_551_MOESM1_ESM.docx]

**Supplementary Information**

**Additional information on methods:** Participants, Working memory task

**Table S1:** Demographic characteristics of the included participants

**Figure S1:** Experimental procedure

**Additional results:** Analysis of the self-reported level of worry across the WM task

**Table S2:** Bivariate correlations

**Additional information on methods**

**Participants**

In the anxious group, two individuals indicated taking psychotropic medication on a stable basis for at least three months (*n* = 1 antidepressant, *n* = 1 benzodiazepine).

**Working memory task**

A verbal WM task called “Memory Updating task” was used to assess WM performance and adapted (Oberauer, Süß, Schulze, Wilhelm, & Wittmann, 2000). The WM task was administered on a 15-inch laptop of different model types using the Java-based platform Tatool (von Bastian, Locher, & Ruflin, 2013). Stimuli were presented in black on a white background. In the task, three or four boxes (called “set size”, representing the level of difficulty) were presented on the screen for 1800ms. In the boxes numbers from 1 to 8 appeared. Participants were asked to remember all presented numbers together with the corresponding box. Next, the presented numbers disappeared and in one of the boxes, an arithmetic operation, included adding or subtracting numbers between 1 and 7, appeared. Participants were asked to recall the previously presented number in the box and to perform the arithmetic operation, type in the result and remember the result for the corresponding box, therefore updating the content of the box. Next, either in the same or in a different box, an arithmetic operation appeared. After nine arithmetic operations (called updating steps), a question mark appeared in each box in random order. Participants were asked to recall the updated content for each box and type it in (called recall run). The instructions were given verbally to the participants and two practice trials were performed before starting the first block. The number of items solved correctly (accuracy) and the reaction time serve as indicators of WM capacity.

In total, each WM block consisted of 150 trials (42 recall trials, 108 updating trials). To standardize the stressful WM task across participants, the two WM blocks were identical; however, the presented numbers and arithmetic operations were different in order to avoid learning effects. The WM task was self-paced and the duration of each block differed between participants.

References

Oberauer, K., Süß, H. M., Schulze, R., Wilhelm, O., & Wittmann, W. W. (2000). Working memory capacity - Facets of a cognitive ability construct. *Personality and Individual Differences*, *29*(6), 1017–1045. https://doi.org/10.1016/S0191-8869(99)00251-2

von Bastian, C. C., Locher, A., & Ruflin, M. (2013). Tatool: A Java-based open-source programming framework for psychological studies. *Behavior Research Methods*, *45*(1), 108–115. https://doi.org/10.3758/s13428-012-0224-y

**Table S1.**

*Demographic characteristics of the included participants*

|  | | **Total sample**  (*n* = 40) | **Group** | |
| --- | --- | --- | --- | --- |
|  | |  | Anxious  (*n* = 26) | Control  (*n* = 14) |
|  | |  |  |  |
|  | |  |  |  |
| **Age** | | 26.4 (7.6) | 27 (8.4) | 25.3 (5.8) |
| **Gender** | |  | | |
|  | male | 20 % | 23 % | 14 % |
| female | | 80 % | 77 % | 86 % |
|  | |  |  |  |
| **Nationality** | |  | | |
|  | CH | 62.5 % | 57.6 % | 71.4 % |
|  | DE | 15 % | 15.3 % | 14.3 % |
| Other | | 20 % | 23 % | 14.3 % |
|  | |  |  |  |
|  | |  |  |  |
| **SES** | | 6.75 (1.3) | 6.9 (1.3) | 6.37 (1.5) |
| **BMI** | | 21.5 (3.4) | 21.5 (2.8) | 21.6 (4.2) |
|  | |  |  |  |
| **Symptom measures** | |  |  |  |
| PSWQ | | 57.7 (11.6) | 64.4 (7.4) | 45.6 (7.1) |
|  | |  |  |  |

*Note*. Means (standard deviation); SES = self-reported socioeconomic status, ranging from 1 (very low) to 10 (very high); BMI = Body mass index; PSWQ = Penn State Worry Questionnaire.

**Figure S1.**

*Experimental procedure*

**
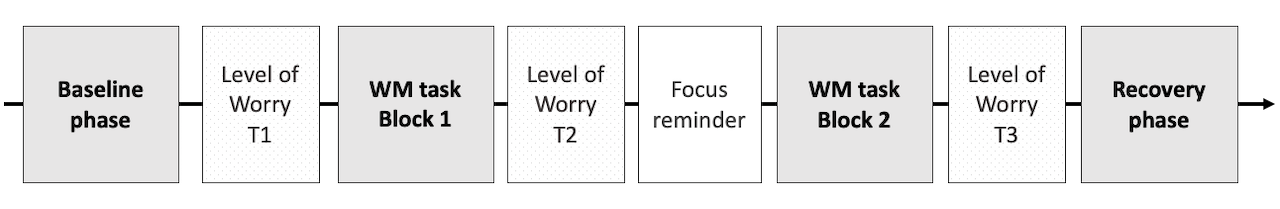
**

*Note.* HRV = heart rate variability; WM = working memory; T1 = before first WM block, T2 = after first WM block T3= after second WM block. For the present study, HRV at Baseline phase, at WM task Block 1 (WM task) and at the Recovery phase are analyzed.

**Additional results**

**Analysis of the self-reported level of worry across the WM task**

To investigate the temporal course of worry before, during and after the stressful WM task, we repeatedly assessed worry using a visual analogue scale ranging from 0 to 100. Descriptively, the highest level of worry in both groups was reported at T1 (Anxious: *M* = 43.6, *SD* = 21.7, Control: *M* = 26.1, *SD* = 18.9). At T2 (in between the WM Blocks), both groups decreased in their perceived level of worry (Anxious: *M* = 37.4, *SD* = 21.6, Control: *M* = 18.1, *SD* = 18.9). At T3, the control groups reported a further decrease in worry level (*M* = 16.2, *SD* = 13.8) whereas the anxious group perceived a higher level of worry (*M* = 40.3, *SD* = 23.2). The anxious group reported higher worry levels at all three time points, compared to the control group. A univariate cross-level interaction model with level of worry as outcome, time (T1, T2, T3) as a level-1 predictor and group as a level-2 predictor revealed significant differences in the perceived level of worry between the anxious and control group at T1 (*t* (37) = 2.53, *p* = .015). The level of worry in the control group at T2 and T3 was not significantly different from T1 (T2: *t* (71) = -1.7, *p* = .09; T3: t (71) = -1.74, *p* = .08) and no significant time x group interactions were found.

**Table S2.**

*Bivariate correlations*

|  |  | **2.** | **3.** | **4.** | **5.** | **6.** | **7.** |
| --- | --- | --- | --- | --- | --- | --- | --- |
| **Cardiovascular indices** |  |  |  |  |  |  |  |
| 1. HR_B | - | -.25 | -.11 | .05 | -.07 | -.04 | .04 |
| 2. HFnu_B |  | - | -.09 | -.22 | -.38* | -.16 | -.29 |
| **Symptom measures** |  |  |  |  |  |  |  |
| 3. PSWQ |  |  | - | .73*** | .47** | .46** | .35* |
| **State worry measure** |  |  |  |  |  |  |  |
| 4. Level of worry (T1) |  |  |  |  | - | .61** | .66** |
| 5. Level of worry (T2) |  |  |  |  |  | - | .83** |
| 6. Level of worry (T3) |  |  |  |  |  |  | - |

*Note.* HR_B = heart rate at baseline; HFnu_B = high frequency heart rate variability in normalized units at baseline; PSWQ = Penn State Worry Questionnaire; T1 = before working memory block 1a, T2 = after working memory block 1b, T3= after working memory block 2b

**p* < .05. ** *p < .01. ***p < .001.*
